# Correction

**DOI:** 10.5195/jmla.2019.664

**Published:** 2019-04-01

**VOLUME 104**

**104(1) January, page 49**

El Sherif R, Pluye P, Gore G, Granikov V, Hong QN. Performance of a mixed filter to identify relevant studies for mixed studies reviews. J Med Libr Assoc. 2016 Jan;104(1):47–51. DOI: http://dx.doi.org/10.3163/1536-5050.104.1.007.

The totals in Table 1, Number of relevant records retrieved and Number of nonrelevant records not retrieved by the filter, should be 1,776 and 1,398, rather than 1,176 and 1,396, respectively. The corresponding total number of records identified in the study should be 4,546, rather than 4,547.

**Corrected Table 1:**
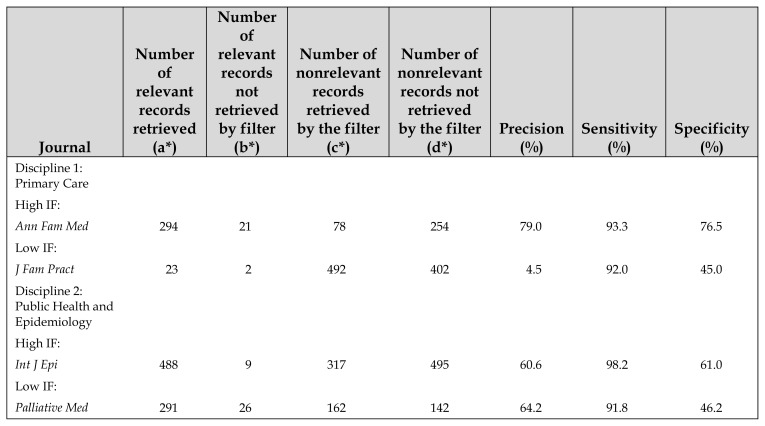

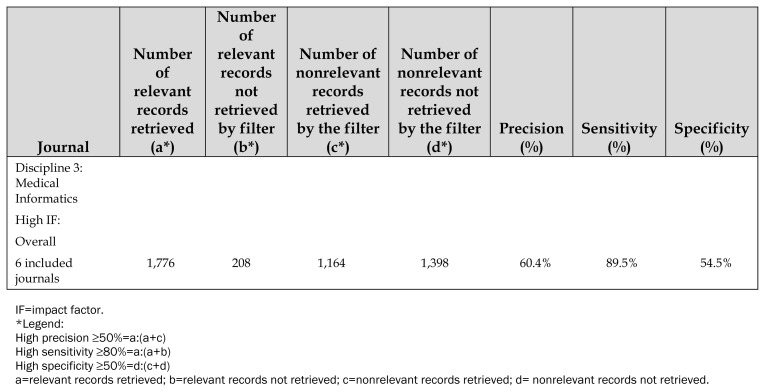
Performance of the mixed filter

Journal	Number of relevant records retrieved (a*)	Number of relevant records not retrieved by filter (b*)	Number of nonrelevant records retrieved by the filter (c*)	Number of nonrelevant records not retrieved by the filter (d*)	Precision (%)	Sensitivity (%)	Specificity (%)
Discipline 1: Primary Care							
High IF:							
*Ann Fam Med*	294	21	78	254	79.0	93.3	76.5
Low IF:							
*J Fam Pract*	23	2	492	402	4.5	92.0	45.0
Discipline 2: Public Health and Epidemiology							
High IF:							
*Int J Epi*	488	9	317	495	60.6	98.2	61.0
Low IF:							
*Palliative Med*	291	26	162	142	64.2	91.8	46.2
Discipline 3: Medical Informatics							
High IF:							
Overall							
6 included journals	1,776	208	1,164	1,398	60.4%	89.5%	54.5%

IF=impact factor.

* Legend: High precision ≥ 50%=a:(a+c) High sensitivity ≥ 80%=a:(a+b) High specificity ≥ 50%=d:(c+d)

a=relevant records retrieved; b=relevant records not retrieved; c=nonrelevant records retrieved; d= nonrelevant records not retrieved.

